# Hybridization promotes color polymorphism in the aposematic harlequin poison frog, *Oophaga histrionica*

**DOI:** 10.1002/ece3.794

**Published:** 2013-10-09

**Authors:** Iliana Medina, Ian J Wang, Camilo Salazar, Adolfo Amézquita

**Affiliations:** 1 Department of Biological Sciences, Universidad de los AndesBogotá, Colombia; 2 Department of Ecology, Evolution and Genetics, Australian National UniversityCanberra, ACT, Australia; 3 Department of Organismic and Evolutionary Biology, Harvard UniversityCambridge, Massachusetts; 4 Facultad de Ciencias Naturales y Matemáticas, Universidad del RosarioBogotá, Colombia

**Keywords:** Admixture, aposematism, coloration, hybridization, microsatellites, phenotypic variation, phylogenetics, poison frogs

## Abstract

Whether hybridization can be a mechanism that drives phenotypic diversity is a widely debated topic in evolutionary biology. In poison frogs (Dendrobatidae), assortative mating has been invoked to explain how new color morphs persist despite the expected homogenizing effects of natural selection. Here, we tested the complementary hypothesis that new morphs arise through hybridization between different color morphs. Specifically, we (1) reconstructed the phylogenetic relationships among the studied populations of a dart-poison frog to provide an evolutionary framework, (2) tested whether microsatellite allele frequencies of one putative hybrid population of the polymorphic frog *O. histrionica* are intermediate between *O. histrionica* and *O. lehmanni*, and (3) conducted mate-choice experiments to test whether putatively intermediate females prefer homotypic males over males from the other two populations. Our findings are compatible with a hybrid origin for the new morph and emphasize the possibility of hybridization as a mechanism generating variation in polymorphic species. Moreover, because coloration in poison frogs is aposematic and should be heavily constrained, our findings suggest that hybridization can produce phenotypic novelty even in systems where phenotypes are subject to strong stabilizing selection.

## Introduction

Understanding the processes that generate genetic and phenotypic variation in the wild is one of the major goals of evolutionary biology (Hoffman and Blouin 2000; Mousseau et al. 2000). There are numerous occurrences of dramatic phenotypic variation among closely related species, in species complexes and among populations within some species (Hoffman and Blouin 2000; Seehausen 2004; Mallet 2005; Wang 2011). Investigating the origins of this diversity provides an opportunity to examine the evolutionary forces underlying biologic diversification (Mousseau et al. 2000; Seehausen 2004; Wang and Shaffer 2008).

In the traditional view, divergent phenotypes arise as a result of novel genetic mutations, and in fact, there are clear examples in which changes in the amino acid sequence of a single locus or even unique mutations in regulatory regions generate major phenotypic modifications (e.g., Bradshaw and Schemske 2003; Hoekstra et al. 2006; Nadeau and Jiggins 2010; Martin and Orgogozo 2013). Additionally, several authors have argued that hybridization may also play an important role in generating biodiversity (Seehausen 2004; Mallet 2005, 2007; Seehausen et al. 2008). In fact, hybridization has proven to be a mechanism capable of generating new traits from existing variation between different species (Grant 1981; Gross et al. 2004; Mallet 2005, 2007; Salazar et al. 2010; Pardo-Diaz et al. 2012; The Heliconius Genome Consortium 2012). When acting together, these two processes, novel mutations and hybridization, can dramatically increase levels of phenotypic variation (Seehausen 2004).

**Figure 1:**
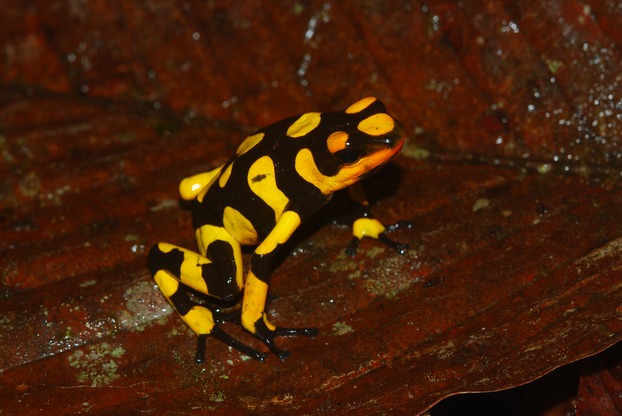
Individual of *Oophaga histrionica* (*sensu lato*) from the Pacific Coast of Colombia. This individual belongs to the population that we call in the text *pHYB*. Photo by Fernando Vargas.

Over 200 species of frogs exhibit some form of color or pattern polymorphism, yet little work has been carried out to investigate selective mechanisms, modes of inheritance, or population-level events giving rise to and maintaining this variation (Hoffman and Blouin 2000). Thus, anuran polymorphisms remain a rich, but largely unexploited system for studying the evolution of phenotypic variation in nature (Hoffman and Blouin 2000; Wang and Shaffer 2008). Several species of poison frogs display dramatic levels of within species phenotypic variation (Noonan and Gaucher 2006; Wollenberg et al. 2008; Noonan and Comeault 2009; Wang and Summers 2010). For instance, four nominal species of harlequin poison frogs that occur in the pacific wet forests of Colombia: *Oophaga histrionica* (Fig. 1, Berthold 1846), *O. lehmanni* (Myers and Daly 1976), *O. occultator* (Myers and Daly 1976), and *O. sylvatica* (Funkhouser 1956), which comprise more than 25 color morphs (Myers and Daly 1976; Lötters et al. 1999; Brown et al. 2011). Aposematic species, such as poison frogs, offer an interesting opportunity to study the evolution of phenotypic variation. Since their aposematic phenotypes are likely subject to strong normalizing selection (Langham 2006; Wollenberg et al. 2008; Noonan and Comeault 2009; Wang 2011) they may be particularly informative with regard to the role of hybridization in increasing phenotypic diversity. For example, if hybridization was found to increase (supposedly constrained) phenotypic diversity in aposematic species, this would suggest that hybridization might be a mechanism capable of contributing even more to the generation of variation in other species with fewer phenotypic constraints.

**Figure 2:**
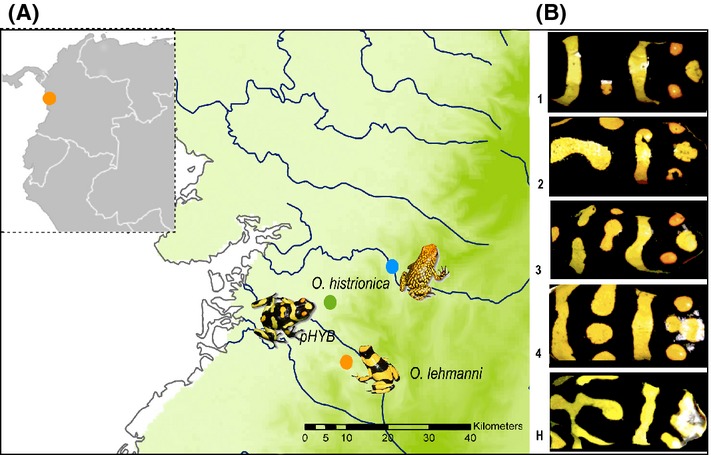
Geographic locations of study populations and their color patterns. (A) Map of the pacific coast of Colombia showing the three study localities: in blue *Oophaga histrionica*, in orange *O. lehmanni,* and in green the *pHYB* population. (B) Examples of color patterns of individuals from the *pHYB* population (1–4) and the pattern from a hybrid between *Oophaga histrionica* and *O. lehmanni* bred in the laboratory (H).

In this study, we investigated the potential occurrence of hybridization between color morphs in poison frogs by examining three divergently colored populations in the *O. histrionica* species complex: one population of *O. histrionica,* one population of *O. lehmanni,* and one putative hybrid population (*pHYB*; Fig. 2A). The harlequin poison frog, *O. histrionica*, is a widespread species with many color morphs, and the red-banded poison frog, *O. lehmanni* (Silverstone 1973), is recognized as a separate species based on call parameters and a unique type of toxin (Daly and Myers 1967), although whether it is distinct from *O. histrionica* is still debated (Lötters et al. 1999). The ranges of these three populations, although probably discontinuous, are separated by less than 15 km (Fig. 2A; Table 1), and crossing experiments between *O. histrionica* and *O. lehmanni* produced, in just one generation, individuals looking like *pHYB* frogs, which appears to have a phenotype that is intermediate between the putative parental populations (Fig. 2B). In closely related *O. pumilio*, experimental crosses have also produced offspring with mixed or intermediate phenotypes (Summers et al. 2004). Thus, these observations suggest that the naturally occurring *pHYB* population may have resulted from hybridization between divergent color morphs. To test this hypothesis, we employed a combination of genetic analysis and behavioral experiments. We first generated an evolutionary framework by reconstructing the phylogenetic relationships between the different color morphs. We then tested the hybrid origin of *pHYB* by comparing allele frequencies of microsatellite markers and levels of historical gene flow with the putative parental lineages. Finally, to test whether the mating behavior of the putative parental populations was consistent with potential hybridization, we tested the mating preferences of females with regard to the coloration and calls of potential male mates.

**Table 1:** Populations (from north to south) employed in the mitochondrial phylogenetic analysis and the microsatellite analysis

Population	Number of individuals for Mitochondrial/Microsatellite analyses	Coordinates (lat, lon)
*Oophaga histrionica-*Arusí	6/0	(5.583, −77.483)
*Oh-*Naranjo	6/0	(3.833, −76.821)
*Oh-*Delfina	20/40	(3.812, −76.692)
*O. lehmanni* (Yellow)	18/24	(3.660, −76.692)
*O. lehmanni* (Red)	4/0	(3.660, −76.692)
*Oh-*Danubio (*pHYB*)	36/40	(4.07, −75.965)
*Oophaga sylvatica*	2/0	(3.683, −76.072)

## Materials and Methods

### Study system and sampling scheme

*Oophaga histrionica* and *O. lehmanni* (Myers and Daly 1976) are both diurnal aposematic frogs that belong to the family Dendrobatidae. Males actively defend territories by uttering advertisement calls; the courtship involves auditory, visual, and tactile signals performed by the male that often lasts several hours, when females are attracted to the males, they approach them and lead them to the laying site (Silverstone 1973; Zimmermann and Zimmermann 1994). Females are responsible of egg attendance and larval transport (Summers et al. 1999a,b). Although some populations of *O. histrionica* include several color morphs, the populations we studied here show very little color variation among individuals. *Oophaga lehmanni* has both yellow and red color morphs; we studied the yellow morph because it occurs in our study area and it was used in the laboratory crossing experiments. Due to serious security issues in this particular region of Colombia, we were unable to fully explore the actual geographic range of each morph. To the best of our knowledge, however, the reproductive individuals are distributed as discrete aggregations of territorial males (colonies) and there is no current contact between any of the studied lineages; the three localities are separated by about 15 km, with no obvious geographic barriers (Fig. 2A; Table 1).

Several crosses were conducted at the Amphibian Laboratory of the Cali Zoo during 2008 (B. Velásquez & A. Amézquita, unpub. data) by placing one female from *O. histrionica* and one yellow male from *O. lehmanni* in the same terrarium until they reproduced. Because tadpoles from these species are obligate oophagous, only six individuals survived to a pre-adult stage where it was possible to detect a color pattern. All of them, however, exhibited coloration that could not be distinguished by eye from the coloration of *pHYB* frogs. We included tissue samples from one of these individuals in our genetic analyses.

To provide a phylogenetic framework, we collected tissue samples via toe clip from a total of 92 individuals from six populations of *O. histrionica, O. lehmanni* and the related species *O. sylvatica* (Table 1). Samples were immediately preserved in 95% ethanol. For the microsatellite analysis, we also collected one of the hybrids between *O. lehmanni* and *O. histrionica* obtained in the laboratory. DNA was extracted and purified using the DNeasy tissue extraction kit (QIAGEN, Valencia, CA), following the manufacturer's protocol; extracted samples were used as template in PCR amplifications.

### mtDNA sequence analysis

We sequenced a region of mitochondrial DNA spanning the cytochrome oxidase subunit 1 (COI) gene and a fragment of 16S ribosomal DNA employing primers COI-A (5′ AGTATAAGC GTC TGG GTA GTC 3′), COI-F (5′CCT GCA GGA GGA GGA GAY CC 3′), 16SA (5′CGCCTGTTTATCAAAAAC3′), and 16SB (5′CCGGTCTGAACTCAGATCACGT3′) to amplify the corresponding gene fragments (Palumbi et al. 1991). Each PCR reaction (25 μL) contained 5–15 ng of genomic DNA, 1.5 μL of primer, and 12.5 μL of Master Mix Taq Polymerase (Promega®, Madison, WI). These regions were chosen because there is already available information on these fragments for other related species (e.g., *O. pumilio*, Hauswalt et al. 2009). Sequencing reactions were performed by Macrogen, Inc (Seoul, Korea). We edited chromatograms and aligned complementary COI and 16S sequences separately in the program Geneious (BioMatters Ltd., Auckland, New Zealand). Genbank accession numbers KF582666-KF582756.

We performed maximum likelihood (ML) phylogenetic analyses (Felsenstein 1981) using PAUP* v4.0b10 (Swofford 2002). Modeltest version 3.6 (Posada and Crandall 1998) was used to choose the most appropriate model by the Akaike information criterion (AIC). We used heuristic searches to estimate the ML phylogenetic tree starting from a neighbor-joining (NJ) tree (Saitou and Nei 1987) with TBR branch swapping. Trees were rooted with *O. pumilio* as an outgroup, with sequences obtained from GenBank (EF597184 for COI and EF597188 for 16S). Statistical support for ML clades was assessed with nonparametric bootstrap analysis (Felsenstein 1985) using 1000 replicates.

We also reconstructed the mtDNA genealogy with a Bayesian optimization criterion (Rannala and Yang 1996; Yang and Rannala 1997) using MrBayes v3.1.2 (Huelsenbeck and Ronquist 2001). MrModeltest v2.3 (Nylander 2004) was used to evaluate and choose one of the potential models of DNA sequence evolution. We conducted one run with five million generations sampled every 1000 generations with four Metropolis-coupled Markov chain Monte Carlo (MCMC) chains using default heating (*T* = 0.2), evaluated the resulting collection of trees for convergence by examining the standard deviation of split frequencies, and discarded all trees obtained before the run achieved a stationary state. We estimated the posterior probability distribution of topologies, branch lengths, and parameter values from the combined 7012 samples of post burn-in trees.

To estimate the degree and direction of mtDNA gene flow between populations, we used the program IM (Hey and Nielsen 2004). We employed a mutation rate of 5.28 × 10^−5^ substitutions per generation with a generation time of 3 years (Noonan and Gaucher 2006), and migration rates (m_1_ and m_2_) were allowed to reach maxima of 2 and 5 migrants per generation. For all data sets, 30 million steps were sampled from the primary chain after a 300,000 burn-in period under the HKY model with 10 chains per set. Mixing properties of the MCMC were assessed by visual inspection of the parameter trend plots, low autocorrelation values between parameters and by examining that the effective sample size (ESS) was higher than 50, as recommended (Hey and Nielsen 2004; Strasburg and Rieseberg 2010). Initial parameters of migration for each pairwise comparison were estimated in DNAsp (Rozas et al. 2003). Each analysis was performed twice to check for consistency.

### Microsatellite genotype analysis

Extracted DNA samples (5–15 ng) were used as template in polymerase chain reactions (PCR) for seven di-, tri-, and tetranucleotide microsatellite loci from Wang and Summers (2009; Dpum14, Dpum63, Dpum44) and Hauswaldt et al. (2010; Oop_B9, Oop_G5, Oop_H5, Oop_E3). Forward primers for each PCR were labeled with a 5′-fluorescent tag (6-FAM, NED, VIC, or PET) for visualization. We amplified loci individually and ran PCR products on an ABI 3730 Genetic Analyzer (Applied Biosystems, Foster City, CA). Fragments were sized with LIZ-500 size standard and collected with GeneMarker v1.90 (Holland and Parson 2011). To confirm that alleles were assigned correctly, we repeated the scoring procedure three times on all samples and also repeated all steps from amplification through scoring on a set of 16 samples. GENEPOP V1.2 (Raymond and Rousset 1995) was used to (1) calculate deviations from Hardy–Weinberg equilibrium (HWE) at each locus using 1000 Markov chain steps and 1000 dememorization steps, and (2) calculate linkage disequilibrium (LD) between all pairs of loci using a likelihood-ratio test with 10,000 permutations. Null alleles frequencies can have an important impact on estimations of population structure; thus, we used FreeNA (Chapuis and Estoup 2007) to correct for their presence in our data set. Levels of population differentiation were calculated with and without null alleles correction using Wright's *F*_ST_Θ estimator (Weir and Cockerham 1984) and their significance (from 10,000 permutations) as is implemented in Arlequin 3.11 (Excoffier et al. 2005). To calculate pairwise migration rates (4N_e_m) between populations, we employed the maximum likelihood coalescent program MIGRATE 3.0.3 (Beerli and Felsenstein 2001). We performed 10 short chains, 4 heated long chains of 30,000 sampled genealogies, with a sampling increment of 50 and a burn-in of 10,000 steps, using uniform priors. Confidence intervals (95%) where established over smoothed posterior distributions.

To assess the overall genetic composition of *pHYB*, we performed a Bayesian model-based clustering algorithm implemented in the program STRUCTURE v2.1.4 (Pritchard et al. 2000). STRUCTURE assumes a model with *K* populations (where *K* is initially unknown), and individuals are then assigned probabilistically to one or more populations (Pritchard et al. 2000). In our case, we used an admixture model with correlated allele frequencies between populations. This model assumes that frequencies in the different populations are likely to be similar, probably due to migration or shared ancestry (Falush et al. 2003). The program was run with a burn-in of 50,000 generations, a Markov chain of 500,000 generations, and from one to four genetic clusters (*K* = 1–4). We determined the number of clusters (*K*) that best describe the data following the method of Evanno et al. (2005), based on the second-order rate of change of the log-likelihood. To confirm the assignment probability analysis from STRUCTURE, we repeated the procedure in the software Instruct with the same parameters. This program also clusters individuals into subpopulations, but it is specifically designed for cases where HWE is not necessarily assumed (Gao et al. 2007). To categorize individual frogs in *O. histrionica*, *O. lehmanni,* and *pHYB*, we ran the program NewHybrids (Anderson and Thompson 2002). This program is a Bayesian method that employs MCMC to calculate posterior probabilities that individuals in a sample belong to parental or hybrid categories (Anderson and Thompson 2002). We used 400,000 sweeps, and model convergence was verified by running four independent chains each of 200 updates and checking homogeneity among the different runs.

To summarize and visualize the microsatellite data, we conducted a Factorial Analysis of Correspondence using the software Genetix v4.5 (Belkhir et al. 2004). This method constructs new variables that summarize the genotypic data by employing operations similar to those used in ordination analyses, but taking into account features typical of genetic data such as homozygosis.

### Sexual selection experiments

To test female sexual preference, we followed Maan and Cummings' (2008) experimental setup (Appendix 1a) with a significant modification regarding females’ reproductive disposition. Females from *O. lehmanni* (*N* = 7) and from *pHYB* (*N* = 7) were collected in the field and kept at the breeding facilities of the Amphibian Lab (Cali zoo) in Colombia. Once *Oophaga* females start to breed under captive conditions, they lay eggs about once a week in a very regular fashion. To increase the probability that tested females were ovulated and thus sexually receptive, and thereby to reduce variation due to female reproductive disposition, we tested in most cases females that had laid eggs at least 1 week before the experiment. In each experimental cage, we placed one female with one male from her own population and one male from one of the other two populations. The experimental arena consisted of a fabric cage with walls covered in 3-cm-thick foam to prevent sound reflection. Experiments were carried out in a darkroom, illuminating the cage with two 75W UV lights, three 50W halogen lights, and two green–blue filters (Lee 728), that simulated natural light conditions (Maan and Cummings 2008). The two experimental males were kept in transparent chambers covered in three of their four sides with 1-cm-thick foam to prevent females from hearing males’ natural calls. As the experiment was meant to test female preference for homotypic males, no matter the kind of information they used, females had both visual cues coming from the males and standardized auditory cues broadcast from (SONY SRS-M30, Tokyo, Japan) loudspeakers at 65 dB (sound pressure level 20 μPa measured at 30 cm). Calls were broadcast from a speaker placed on top of each male's chamber; these calls were representative of that of the male's population.

To provide replicated auditory stimuli that mimicked the natural calls at each locality, we synthesized five advertisement calls per population using temporal parameters within 0.5 SD of the population averages. This was carried out to control and account for most of the variation inside the population (following Amezquita et al. 2006). Peak frequency is correlated with body size in many frog species (Gerhardt 1994); therefore, to avoid size-biased preferences by females, we used the average value of peak frequency for all calls of the same population (Appendix 1b). The auditory stimuli were synthesized on Audacity Sound File Editor v1.2.4 (http://audacity.sourceforge.net/).

Females were placed in the center of the experimental arena while the male chambers were covered with foam. As acclimation time, females were allowed to roam freely for at least 20 min or until they were not trying to escape. After removing visual barriers between male chambers and the experimental arena, we turned on the speakers and the two auditory stimuli were broadcasted as antiphonal series of 50 calls separated by silent intervals of 15 sec. We then recorded female behavior for 50 min, although, in most cases, a preference was detectable within 20 min. We counted the number of times each female entered an interaction zone (IZ) with each male and the duration of each visit. The IZ was defined as a 7 cm (about twice female body length) square in front of each male chamber (Maan and Cummings 2008). The main experiment tested whether *pHYB* females preferred homotypic males against heterotypic (*O. histrionica* or *O. lehmanni*) males. We also report results for a second experiment to test whether *O. lehmanni* females shared a similar pattern of mate choice.

To quantify mate choice, we calculated relative probabilities of preference following the maximum likelihood model employed by Jiggins et al. (2001) and Merrill et al. (2011). Briefly, we maximized the function ln(*L*) = ∑ *m*_*i*_ ln (*P*_*j*_ + *n*_*i*_ ln (1 – *P*_*j*_)), where *m*_*i*_ is the amount of time the female *i* spent in the IZ of the *m* type male and *n*_*i*_ is the amount of time the female *i* spent in the IZ of the *n* type male. *P*_*j*_ is the probability of females of phenotype *j* responding with behaviors directed toward the *m* type of males. The analysis was run using the solver option in EXCEL (Microsoft, Redmond, WA). Support limits, asymptotically equivalent to 95% confidence intervals, were obtained by searching for values that decreased ln(*L*) by two units. We used this approach because is much more robust than conventional statistics given our limited number of experimental subjects.

We used a likelihood-ratio test (LRT = −2ln Δ(*L*)), which follows a chi-square distribution, to test whether the probability of response toward the heterotypic male differed from the probability of response toward the homotypic male, and whether the probability of *pHYB* females preferring *pHYB* males depended on the combination of males.

## Results

### mtDNA sequence analysis

**Figure 3:**
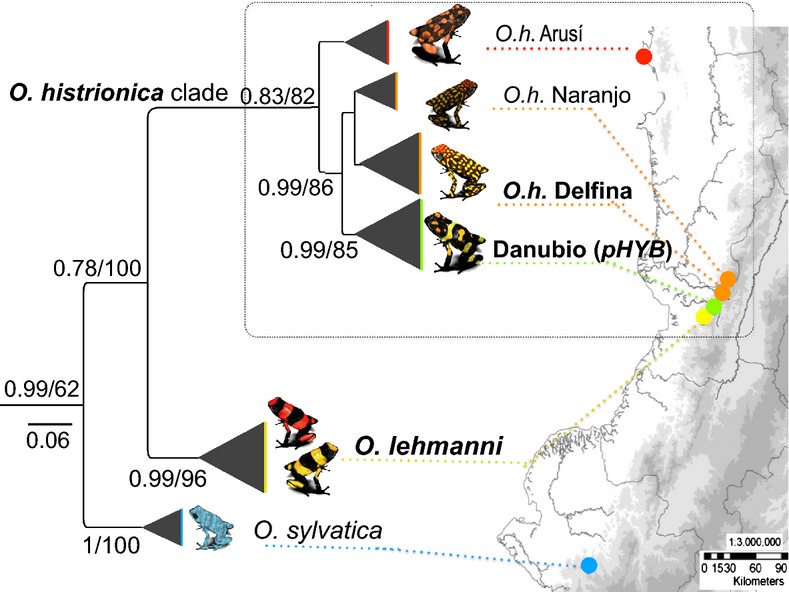
Phylogenetic reconstruction of relationships between populations of *Oophaga histrionica* as inferred by maximum likelihood and Bayesian analysis from COI mtDNA fragments and their geographic locations. Bayesian posterior probabilities and bootstrap values are shown above each branch. Triangle size is proportional to the number of individuals sequenced. The square highlights the *Oophaga histrionica* clade. Populations studied in this work are highlighted.

The general time reversible model (GTR+I) was identified as the optimal model of sequence evolution for our data set; therefore, it was used in all subsequent phylogenetic analyses. The phylogenetic relationships found under ML and Bayesian reconstructions show six well-differentiated clades (Fig. 3). Both reconstructions showed the red and yellow morphs of *O. lehmanni* as a monophyletic group (posterior probability = 0.99, ML bootstrap = 96). Haplotypes from *pHYB* constituted a well-supported monophyletic group (posterior probability = 0.99, ML bootstrap = 85) sister to haplotypes from *O. histrionica* (Delfina), its putatively parental lineage, and *O. histrionica* (Naranjo; posterior probability = 0.99, ML bootstrap = 86). These three populations were moderately supported as monophyletic with the remaining northern population of *O. histrionica* (Arusí; posterior probability = 0.83, ML bootstrap = 82).

Migration estimates from IM (Table 2) revealed that historical gene flow occurred among *O. histrionica*, *O. lehmanni,* and *pHYB* (mean migration rate = 0.4; Table 2; Appendix 2a). The migration rate exceeded one migrant per generation in only one case, from *O. histrionica* to *pHYB* (migration rate = 1.09). Although these estimates were consistent across different runs, the information is based in just one fragment of the mitochondrial genome, and interpretations based on this sole result must be carried out cautiously.

**Table 2:** Migration estimates (migrants per generation) for each pair of populations in both directions as inferred from mitochondrial data. Numbers in parenthesis indicate 90% confidence intervals. The number of migrants exceeded one migrant per generation in just one case (from *Oophaga histrionica* to pHYB)

From/To	*O. histrionica*	pHYB	*O. lehmanni*
*O. histrionica*		1.09 (1.2–4.20)	0.41 (0.3–4.04)
pHYB	0.001 (0.0025–0.84)		0.11 (0.002–2.45)
*O. lehmanni*	0.28 (0.002–2.04)	0.51 (0.2–3.33)	

### Microsatellite genotype analysis

**Figure 4:**
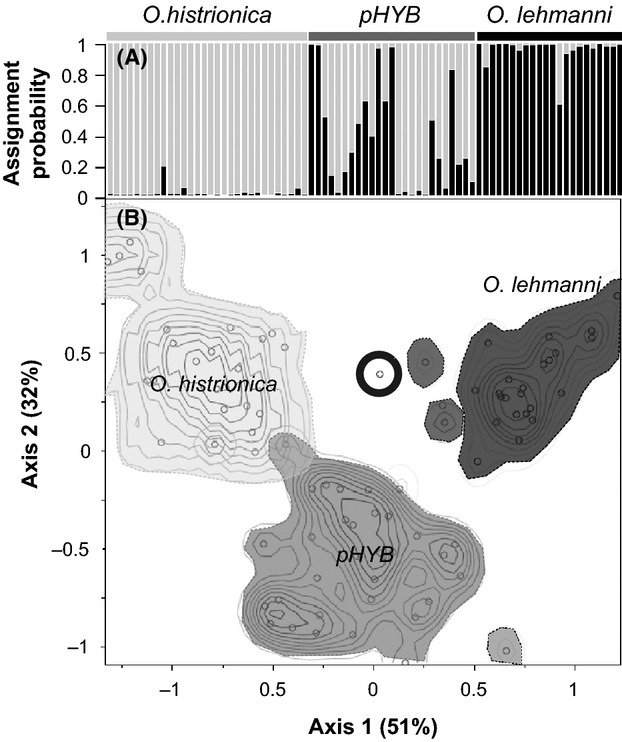
Results of allelic variation analyses inferred from microsatellite data. (A) Bar plots showing Bayesian assignment probabilities for individual frogs as inferred by Instruct for two clusters. The proportion of each bar that is black or gray represents the probability of belonging to the cluster from *O. lehmanni* or *O. histrionica*, respectively. (B) Bidimensional plot of the factorial correspondence analysis. Each dot represents an individual, and each axis contains information from the allele frequencies. Density clouds were calculated in JMP and correspond to nonparametric confidence intervals at 5% steps. The dot circled in black represents a hybrid between *Oophaga histrionica* and *O. histrionica* bred in the laboratory.

The seven microsatellite loci exhibited between 10 and 23 alleles per locus. The linkage disequilibrium analysis showed significant correlation between loci Dpum44 and Oop_B9 (*P* < 0.001); therefore, we employed just Oop_B9 in subsequent analyses. The locus Dpum44 showed significant heterozygote deficit for *pHYB* in the HW test (*P* < 0.001, df = 14), and the remaining loci did not show HW equilibrium deviations in any of the three populations. *F*_ST_ estimates indicated significant differentiation between *O. lehmanni* and *O. histrionica* (*F*_ST_ = 0.107, *P* < 0.001) and between *pHYB* and *O. histrionica* (*F*_ST_ = 0.057, *P* < 0.001) but not between *pHYB* and *O. lehmanni* (*F*_ST_ = 0.034, *P* > 0.05). The results inferred by STRUCTURE, Instruct, and NewHybrids were highly consistent; we report only the Instruct results. Interestingly, NewHybrid classified individuals from *pHYB* as *F*_2_ genotypes (*P* > 80% for *F*_2_ vs. *P* = 0 for the other categories; Appendix 2b), something that has not yet been tested in the laboratory. Following Evanno et al. (2005), the most likely number of clusters in both STRUCTURE and Instruct was *K* = 2 (LnP(D) = −2161.59, Delta *K* peak = −60.71, Appendix 2c). Assignment probabilities rendered two well-structured clusters (*O. histrionica* and *O. lehmanni*) and indicated that each individual from the *pHYB* population typically showed some probability of being assigned to either cluster, suggesting that the *pHYB* population shares allele frequency variation with *O. histrionica* and *O. lehmanni* (Fig. 4A). When assuming *K* = 3 (LnP(D) = −2242.33) and/or a model with no admixture, *pHYB* did not constitute a well-differentiated genetic cluster (Appendix 2d), suggesting, again, that *pHYB* shares allele frequency variation with *O. histrionica* and *O. lehmanni*. The historical gene flow analyses in the nuclear data revealed that although there is an overall genetic contribution from both, putative parental populations to *pHYB*, the highest levels of migration occur from *O. lehmanni* to *pHYB* (number of migrants: 8.48, Table 3). Consistently with the gene flow pattern in mtDNA, migration rates from *pHYB* to *O. histrionica* and *O. lehmanni* were lower than the ones observed in the opposite direction (Tables 2 and 3).

**Table 3:** Migration estimates (migrants per generation) for each pair of populations in both directions as inferred from microsatellite data in MIGRATE. Numbers in parenthesis indicate 95% confidence intervals

From/To	*Oophaga histrionica*	pHYB	*Oophaga lehmanni*
*O. histrionica*		4.68 (1.04–6.54)	5.14 (2.16–7.26)
pHYB	1.04 (0.85–1.24)		2.51 (0.58–4.25)
*O. lehmanni*	3.83 (1.89–5.56)	8.48 (8.34–13.06)	

The factorial analysis of correspondence rendered two main axes that explained most of the genotypic variation (83%) exhibited by the microsatellite markers (Fig. 4B). In the *x*-axis projection, the *pHYB* population is intermediate between *O. histrionica* and *O. lehmanni*, a pattern consistent with the hybridization hypothesis. Additionally, *pHYB* is placed as an independent cluster, distinguishable from the others. Moreover, we were able to analyze the same microsatellite markers for one of the hybrids reconstructed in the laboratory, which was also placed in an intermediate position in the *x*-axis.

### Sexual selection experiments

**Figure 5:**
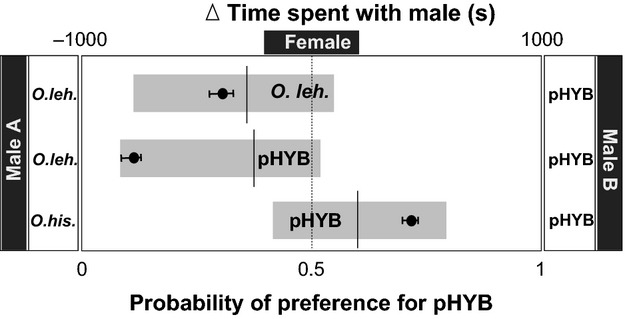
Results of female mate-choice experiments for three experimental groups. Black dots with confidence intervals represent the preference probability according to the maximum likelihood model. Gray boxes represent minimum and maximum time spent with one male or another. Vertical lines through the boxes represent average times. Names in the center represent tested females.

Overall, the female preference for homotypic males depended on the combination of available males (*G*_2_ = 922.5, *P* < 0.001; Fig. 5). *pHYB* females strongly preferred *O. lehmanni* males (mean percentage of time = 85%, SD = 18%) over homotypic males (*G*_1_ = 1940.92, *P* < 0.001). On the other hand, they preferred homotypic males (mean percentage of time = 74%, SD = 23%) over *O. histrionica* males (*G*_1_ = 804.92, *P* < 0.001). When *O. lehmanni* females were exposed to their conspecifics and *pHYB* males, they preferred *O. lehmanni* males (*G*_1_ = 453.62, *P* < 0.001).

## Discussion

Recent theory has suggested that hybridization can potentially affect the evolution of phenotypic diversity under a variety of conditions, and genetic studies demonstrate that one of the outcomes is the interchange of pre-existing adaptive traits between species (Anderson et al. 2009; Song et al. 2011; Pardo-Diaz et al. 2012; The Heliconius Genome Consortium 2012). However, especially for the most phenotypically diverse species and species complexes, hybridization may have played an important role in increasing variation by producing novel phenotypes from crosses between existing, divergent phenotypes (Kim et al. 2008; Salazar et al. 2010; Pardo-Diaz et al. 2012). In the case of the *O. histrionica* complex of poison frogs in Colombia, our results support a role for hybridization in creating new phenotypic variation among the three lineages studied here.

Our analyses of population structure and genetic correspondence unambiguously support the hybridization of parental genotypes in *pHYB*. Variation in microsatellite alleles was best divided into two clusters (*O. lehmanni* and *O. histrionica*), and *pHYB* individuals shared some allele variation with each of them (Fig. 4A). This pattern is highly consistent with other reports of putative hybridization (e.g., Gompert et al. 2006; Larsen et al. 2010). Although *pHYB* appears as an independent, intermediate cluster in the genetic factorial analyses (Fig. 3B), our estimates of population differentiation (*F*_ST_) between *pHYB* and the parental lineages are fairly low (*F*_ST_ = 0.034 with *O. lehmanni* and *F*_ST_ = 0.057 with *O. histrionica*), compared with differentiation between the two species (*F*_ST_ = 0.107). We also detected higher rates of mtDNA migration from *O. histrionica* (*N*_M_ = 1.09) and *O. lehmanni* (*N*_M_ = 0.513) into *pHYB* than between the other population pairs. Furthermore, numbers of migrants detected from *O*. *histrionica* and *O. lehmanni* to *pHYB* were higher than those observed in the opposite direction in nuclear data (Table 3). Both results suggest that there has been admixture. Interestingly, distinct genetic markers revealed slightly different genetic contribution from the parental species to *pHYB*. Thus, mtDNA topology and migration rates suggest a hybrid population with mtDNA from *O. histrionica*, whereas microsatellites showed a remarkable historical migration from *O. lehmanni* toward *pHYB,* although these loci also indicated that hybrids are sharing alleles with both parental populations (Fig. 4 and Appendix 2b). Additionally, although there is considerable genetic differentiation between *O. lehmanni* and *O. histrionica*, our data do not support or reject the specific status of *O. lehmanni*. In any case, the *pHYB* population appears to be both phenotypically and genetically intermediate between the two putative parental populations, suggesting that this novel phenotype has arisen through hybridization.

In concordance with our genetic data, the results of our behavioral experiments also support the occurrence of hybridization between the putative parental populations. Hybridization events should be inextricably linked to mating behavior and mate preferences (Mallet 2005). Hybridization is unlikely when individuals exhibit very strong assortative mating via mate recognition signals (Summers et al. 1999a,b; Mallet 2005, 2007). Previous studies on *O. pumilio* (Reynolds and Fitzpatrick 2007; Maan and Cummings 2008) revealed a trend for assortative mating based on coloration, which is only partly supported here. Females in our study system prefer homotypic or heterotypic males depending upon the combination of available males. Indeed, in a parallel mate choice study conducted at a larger scale (Velásquez & Amézquita, unpublished data), *O. histrionica* and *O. lehmanni* females more often preferred differently colored (heterotypic) over homotypic males. These behavioral data provide a plausible mechanism behind hybridization events: given certain combination of available males (e.g., in a contact zone between two species/morphs), females might be attracted to heterotypic males, which should increase chances of hybridization. Hybrid females, in turn, might either maintain ongoing introgression from one of the parental lineages or show assortative mating, depending on the combination of available males. Given the close geographic proximity of many populations and the occurrence of several populations with apparently intermediate phenotypes, it is plausible that hybridization between divergent phenotypes may also be occurring among other populations in *O. histrionica*.

Finally, the pattern of female preference we observed (pHYB females prefer *O. lehmanni* males) is also consistent with results from our mtDNA phylogenetic analysis, which rendered *pHYB* nested within *O. histrionica*. Because mtDNA is maternally inherited, the tree topology is compatible with sexually asymmetrical gene flow. If gene flow occurs primarily between *pHYB* females and *O. lehmanni* males, then *pHYB* mtDNA should still appear most closely related to *O. histrionica* and will show low signs of mitochondrial introgression from *O. lehmanni*. Likewise, the nuclear microsatellite genetic information will show *pHYB* as an admixture between *O. histrionica* and *O. lehmanni*, which indeed happened here. Thus, both the genetic and behavioral evidence suggest a pattern of asymmetric hybridization between the two lineages we studied. It is unlikely that *pHYB* is the result of a transient hybridization swarm between the putative parental species (i.e., “short-lived hybrids” generated by well-established species) given the presence of incipient mating preferences, geographic discontinuity, and genetic divergence of these morphs. However, it is still necessary to establish the entire distribution of these frogs in order to make a comprehensive interpretation of their hybridization pattern. Nonetheless, better security conditions in Colombia are needed to address this issue.

Hybridization in animal systems is not uncommon in nature and contributes to share and create phenotypic diversity. However, the evolutionary dynamics producing a newly adapted genetic pool after hybridization are largely unknown (Nolte and Tautz 2010). Our study provides the first evidence that hybridization coupled with context-dependent female preferences can contribute to the evolution of phenotypic variation in polymorphic aposematic frogs. Of course, hybridization events can only occur after other mechanisms have generated initial variation and led to divergence, but we propose that hybridization can be a mechanism that may explain part of the huge variation observed in these polymorphic poison frogs. Future studies should investigate in detail how selective mechanisms interact and under which circumstances they promote the persistence of novel phenotypes in various species.

## Data Accessibility

Data deposited in the Dryad Repository: http://doi.org/10.5061/dryad.rb75g

## Appendix 1

(a) Mate-choice experimental setup designed to test female sexual preference. Each corner has a chamber with a male inside and a speaker with a synthetic call that corresponds to the male's population.

(b) Acoustic parameters employed in the synthesis of the stimuli used in mate-choice experiments for each of the populations of the frogs *O. histrionica*, pHYB, and *O. lehmanni*. For temporal parameters, dark bars correspond to pulse duration and light bars correspond to silence duration. For spectral parameters, colors from dark to light correspond to initial, middle, and final frequencies, respectively.

## Appendix 2

(a) Distribution of migration probabilities between populations as calculated in the program IM for mitochondrial data.

(b) NewHybrid results from microsatellite data. Bars show number of individuals per population assigned to different categories (pure *O. histrionica*, pure *O. lehmanni,* and the *F*_2_ hybrid *pHYB*).

(c) Graph showing difference in the likelihood of the number of clusters according to Evanno's method. Likelihood values taken from the Instruct analysis.

(d) Individual posterior probabilities of assignment calculated when *K* = 3 in the program Instruct. Each bar represents an individual, and the height of each color segment represents the probability of assignment to that genetic cluster. Assuming a third genetic cluster does not result in individuals from the *pHYB* population being assigned to a unique cluster rather than showing mixed probabilities of assignment to multiple clusters.
